# Scleral Buckling Versus Sutureless Parsplana Vitrectomy in the Management of Primary Rhegmatogenous Retinal Detachment

**DOI:** 10.7759/cureus.11579

**Published:** 2020-11-19

**Authors:** Padmaja K Rani, Raja Narayanan, Riddhima S Deshpande, Divya Balakrishnan, Mohammad H Ali

**Affiliations:** 1 Vitreoretinal Diseases and Teleophthalmology, LV Prasad Eye Institute, Hyderabad, IND; 2 Vitreoretinal Diseases, LV Prasad Eye Institute, Hyderabad, IND; 3 Vitreoretinal Diseases, LV Prasad Eee Institute, Hyderabad, IND; 4 Biostatistics, LV Prasad Eye Institute, Hyderabad, IND

**Keywords:** rhegmatogenous retinal detachment, scleral buckling, sutureless pars plana vitrectomy

## Abstract

Purpose

The aim of the article is to compare scleral buckle (SB) and primary sutureless pars plana vitrectomy (PPV) without SB in rhegmatogenous retinal detachment (RRD) repair.

Methods

A retrospective study of rhegmatogenous RD surgeries performed between eyes with proliferative vitreoretinopathy (PVR) up to grade B and a minimum of two months postoperative follow-up were included. The primary outcome measure was an improvement in the final best-corrected visual acuity (BCVA) and secondary outcome measures were a final anatomical success, number of resurgeries, and cataract progression.

Results

A total of 37 eyes in the SB group and 30 eyes in the sutureless PPV group were included. The mean follow-up was 7.5 ± 5 months and 9 ± 4 months in the SB and PPV group, respectively. The improvement in the final BCVA from baseline was four lines in the SB group and five lines in the PPV group (p=0.87). The final anatomical success was 97% in SB and 93% in the PPV group. The number of re-surgeries for attachment of retina were higher in the PPV group, (SB: 8/37 vs PPV: 8/30 p=0.03).The number of resurgeries (16/37 vs. 33/30; p=<0.05), cataract progression (3/37 vs. 10/30; p=0.01), and the mean number of hospital visits (6 vs. 9; p=0.001) were significantly higher in the sutureless PPV group.

Conclusions

Visual acuity improvement and anatomical success rates were similar between SB and sutureless PPV in RRD repair. The number of operations, cataract progression, and the mean number of hospital visits were higher in the sutureless PPV group.

## Introduction

Rhegmatogenous retinal detachment (RRD) is an important cause of visual impairment. Surgical management is the gold standard of care for RRD. Various surgical modalities like scleral buckling (SB), pneumoretinopexy, pars plana vitrectomy (PPV) with tamponade have been tried in cases with RRD based on its complexity. Significant evidence (in prospective and retrospective studies) comparing PPV vs. PPV+SB (encircling band) in the management of primary RRD is available [1-4]. Evidence from a randomized clinical trial comparing SB and 20-gauge PPV suggests that scleral buckles have favorable anatomical and functional success in phakic eyes with RRD, and PPV is better in pseudophakic eyes with RRD [1].

Previously, SB was considered as standard therapy in the repair of primary RRD [5, 6]. However, with the recent advances in vitreoretinal techniques and instrumentation, there is a trend towards sutureless vitrectomy to manage primary RRD. Faster visual recovery, safety, and efficacy of sutureless PPV in the management of primary RRD have been established through various studies [7, 8]. However, there is a paucity of information about the direct comparison of SB and sutureless PPV for primary RRD. The present study aims to compare the outcomes of SB and sutureless PPV in RRD repair.

## Materials and methods

This is a retrospective study of rhegmatogenous RD surgeries performed over a one-year period at the LV Prasad Eye Institute (LVPEI). The Institutional Review Board approved the retrospective analysis of charts for the study, and all the procedures adhered to the tenets of the Declaration of Helsinki. The data collected included ocular history, demographics, baseline visual acuity, a complete ocular exam including the intraocular pressure (IOP) at each visit, the surgeries performed, the final visual acuity, and the follow-up duration. The extent of RD, location of breaks, the extent of PVR was noted. Resurgeries for recurrent RD as well as cataract surgery, silicone oil removal were noted.

Patients with proliferative vitreoretinopathy (PVR) up to grade B and subjects with a minimum of two months postoperative follow-up were included. Subjects with comorbid eye diseases that can affect visual acuity like glaucoma, macular hole, PVR more than grade B, cases of infectious retinitis, or tractional/exudative/combined RD were excluded.

The primary outcome measure was best corrected visual acuity (BCVA) improvement, and the secondary outcome measures were baseline features, anatomical success, number of operations, cataract progression, and cost-effectiveness analysis between SB vs. sutureless PPV groups.

In the scleral buckling procedure, all causative retinal breaks for RD were localized, retinopexy in the form of cryotherapy was done to the retinal breaks, and a segmental scleral buckle of appropriate width was placed based on the location of retinal breaks. Drainage of subretinal fluid was performed based on the surgeon's discretion.

In sutureless PPV, 23 or 25g sclerotomies were placed, and a standard PPV was performed. All retinal breaks were cauterized, and after fluid air exchange, endolaser photocoagulation was performed to all retinal breaks in primary surgeries. In eyes undergoing resurgeries, 360 degrees barrage laser was done to the peripheral retina. Tamponade, in the form of gas/silicone oil (1000 Cst), was done based on the surgeon's discretion.

All the graphs were performed made using GraphPad Prism (GraphPad Software, version 6.00 for Windows, La Jolla, USA). Independent samples t-test and chi-square tests were used as appropriate. P-value <0.05 was considered statistically significant.

## Results

A total of 37 eyes in the SB group and 30 eyes in the sutureless PPV group that met the eligibility criteria were compared. Table 1 shows the baseline features among the SB and sutureless PPV groups. Subjects in the SB group compared to those in the sutureless PPV group were found to be phakic, younger, with longer duration of RD, and with more number of inferior breaks. However, the mean presenting visual acuity was similar in both groups.

**Table 1:** Comparison of baseline features between SB and sutureless PPV groups SB - scleral buckle; PPV - pars plana vitrectomy; RD - retinal detachment; VA - visual acuity; IOP - intraocular pressure

Variable	SB (n=37)	Sutureless PPV (n=30)	p
Male : female	29 : 8	17 : 13	0.07
Mean age	26.4 SD 13.77	48.6 SD 15.21	0.0001
Duration of RD (days)	85.7	11.1	0.03
Mean number of breaks	1.7	1.1	0.009
Distribution of breaks (superior/inferior/temporal)	Sup 9 Inf 22	Sup 18 Inf 10	0.01
VA	0.94, 20/160	1.0, 20/200	0.87
IOP	12.27	12.93	0.53
Phakic status	34	17	0.001

Table 2 shows a comparison of functional and anatomical outcomes between SB and sutureless PPV groups.

**Table 2:** Comparison of outcomes between SB vs. sutureless PPV groups SB - scleral buckle; PPV - pars plana vitrectomy; VA - visual acuity; IOP - intraocular pressure

Variable	SB (n=37)	Sutureless PPV (n=30)	p
IOP			
1 day IOP	17.15	14.89	0.08
1 week IOP	13.4, 1 High	22.3, 9 High	0.0002
1 month IOP	16.51, 5 high	18.75, 5 high	0.35
3 month IOP	14.0	16.4, 2 high	0.58
VA			
1 week VA	20/125	20/200	
1 month VA	20/100	20/100	
3 months VA	20/80	20/80	0.87
Resurgery rate	8/37	8/30	0.003
Cataract Progression	3	10	0.01
Mean no. of visits	6	9	0.0014
Final anatomical success	1/37 detached (97% attached)	2/30 detached (93% attached)	0.58

The primary outcome measure, the improvement in best corrected Snellen’s visual acuity, was 4 lines in SB and 5 lines in the PPV group. The final anatomical success was 97.29% (36/37) in the SB group and 93.33% (28/30) in the sutureless PPV group. The primary anatomical success was 79% (29/37) in SB and 73% (22/30) in the PPV group. The number of resurgeries for retina attachment between SB vs. PPV was (8/37 vs. 8/30 p=0.03) higher in the PPV group. The mean IOP showed an initial high spike trend, especially in the first week in the sutureless PPV group compared to the SB group (22.3, 9 having high IOP>21 vs. 13.4, 1 having IOP>21). This change in IOP stabilized by the first month in both the groups. Among the sutureless PPV group, 21 subjects had silicone oil (1000 Cst) tamponade, and nine had gas tamponade with C3 F8.

The number of resurgeries for attachment of retina between SB vs. sutureless PPV was 8/37 vs. 8/30 (p=0.03), cataract progression was 3/37 vs. 10/30 (p=0.01), the mean number of hospital visits was 6 vs. 9 (p=0.001) being significantly higher in the sutureless PPV group. The number of resurgeries was significantly higher in the sutureless PPV than the SB group (SB 16/37 vs. PPV 33/30; p<0.05). Amongst the 16 resurgeries in the SB group, eight eyes underwent a resurgery for retinal attachment (seven eyes had vitreoretinal surgery with silicone oil implantation and one eye had gas injection), seven eyes underwent silicone oil removal, and one eye had resurgery with oil implantation. Among 33 resurgeries in the PPV group, eight eyes underwent resurgery for retinal attachment (eight eyes with membrane peeling, endolaser, and silicone oil exchange), 15 eyes underwent silicone oil removal, seven eyes underwent cataract surgery, one eye underwent intraocular lens explantation with anterior chamber (AC) IOL implantation and two eyes underwent vitreous biopsy and intraocular antibiotic injection. At the last follow-up, one eye in the SB group and three eyes in the PPV group had silicone oil. One patient in the PPV group (both eyes) developed endophthalmitis following oil removal and underwent vitreous biopsy with intraocular antibiotic injections. The organism was suspected to be fungal based on a polymerase chain reaction. In one eye, the same patient had developed redetachment after silicone oil removal after the resolution of endophthalmitis. Logistic regression analysis showed no statistically significant influence of age, phakic status, and inferior break location on the final anatomical outcome between SB and PPV groups. A total of 5/30 eyes in the PPV group had silicone oil-induced glaucoma, which did not settle after silicone oil removal (SOR). All were managed medically. One patient had silicone oil-induced glaucomatous optic atrophy.

## Discussion

In the present study, important RRD repair outcomes, which are a visual recovery (20/80) and anatomical success (retinal attachment rate 97% vs. 93%), were similar between both the SB and the sutureless PPV groups. Recently, in a study by Rush et al. comparing sutureless PPV with scleral buckling and also PPV with scleral buckling in medium complex RRD, the overall primary anatomic success rate was 83.6% (95% confidence interval, 80.3%-86.5%) in all three groups. Logistic regression analysis did not demonstrate a significant difference between the three techniques regarding the likelihood of anatomic success or the likelihood of achieving a Snellen BCVA of 20/40 or better [9]. 

In our study, patients in the SB group were phakic, of a younger age group, with a longer duration of RD, and with more number of inferior breaks. These subjects achieved significant visual recovery as well as excellent anatomical success comparable to sutureless PPV. The phakic status and location of inferior breaks associated with favorable anatomical and functional success were noted in the SB group in both the prospective and retrospective studies [1-4, 10-12].

Our earlier study showed the efficacy and safety of sutureless PPV in RRD repair [7]. The present study also found that visual recovery and anatomical success rates following sutureless PPV are satisfactory. Case selection is the key factor in achieving these visual and anatomical outcomes. Cataract progression was significantly more in the PPV group in comparison to the SB group. A similar progression of cataract was noted in both the prospective as well as retrospective studies [1, 10, 13].

The mean IOP showed an initial high spike trend, especially in the first week in the sutureless PPV group compared to the SB group (22.3, 9 having high IOP>21 vs. 13.4, 1 having IOP>21). This change in IOP stabilized by the first month between both the groups. The initial spikes, especially in the first week in the PPV group, necessitated the use of anti-glaucoma medications adding to the cost of eye care for the patient.

The average cost per procedure in the SB group is $400 and in the PPV group is $1000. We collected information on the number of hospital visits with SB vs. PPV. We used the mean number of hospital visits, the number of resurgeries as indicators of costs incurred by the patient. The mean number of hospital visits (9 vs. 6) and the number of resurgeries were significantly higher in the PPV group compared to the SB group. To the best of our knowledge, our study is the first to provide information on the cost comparison between SB and PPV for RRD, a factor that is to be considered while deciding the surgical modality in RRD repair.

The limitations of our study include the retrospective nature of the study and the small sample. The study sample is not homogenous for precise comparison as the presenting features of RRD are too varied to achieve such uniformity. Another important limitation is a selection bias as cases are selected for SB/PPV as per the suitability of intervention.* * Hence, we have highlighted the various differentiating morphological features of RRD between the two groups.

## Conclusions

To conclude, both SB and sutureless PPV have an important role in managing primary RRD repair. However, the PPV group has a higher number of operations, cost, cataract progression, and the mean number of hospital visits, the anatomical and visual outcomes of PPV are almost comparable to SB, which is a relatively simple procedure compared to PPV.
